# Monomicrobial Gram-Negative Necrotizing Fasciitis of the Lower Extremity

**DOI:** 10.7759/cureus.75298

**Published:** 2024-12-07

**Authors:** Saba Suleman, Bryan Youree, Shovendra Gautam

**Affiliations:** 1 Internal Medicine, Baylor Scott and White All Saints Medical Center, Fort Worth, USA; 2 Infectious Diseases, Baylor Scott and White All Saints Medical Center, Fort Worth, USA; 3 Internal Medicine, Baylor Scott and White Health, Fort Worth, USA

**Keywords:** e. coli, gram-negative infections, lower extremity cellulitis, lower extremity swelling, necrotizing fasciitis, necrotizing soft tissue infections

## Abstract

Unlike other skin and soft tissue infections, necrotizing fasciitis (NF) is a rare and potentially life-threatening condition. It is usually caused by polymicrobial infections or monomicrobial gram-positive organisms, mainly *Staphylococcus* and *Streptococcus*. Monomicrobial gram-negative* Escherichia coli *(*E. coli*) NF is a rare form of NF, primarily reported in patients with underlying comorbidities or immunocompromised states. Here, we present a novel case of monomicrobial gram-negative NF caused by *E. coli *in an otherwise healthy patient. This case highlights that *E. coli* NF cannot be solely attributed to comorbidities, and physicians should be aware that NF can occur from *E. coli *infection in the absence of underlying health conditions.

## Introduction

Necrotizing fasciitis (NF) is a rapidly progressing soft tissue infection characterized by spread along the fascial plane and extensive tissue necrosis [1]. It necessitates prompt surgical debridement, as bacteria typically enter through breaks in the skin, such as cuts, burns, insect bites, puncture wounds, and surgical incisions [1,2]. Despite antibiotic treatment and surgical intervention, NF remains a severe, life-threatening condition with a mortality rate exceeding 15%. Factors influencing mortality include age, underlying health conditions, immunocompromised states, and the severity of the infection [1,3].

NF is taxonomically differentiated into three distinct types. Type 1 is a polymicrobial infection involving a mix of aerobic and anaerobic bacteria. Type 2 is a monomicrobial infection, typically caused by either Group A *Streptococcus* or *Staphylococcus aureus*. Type 3 is associated with *Vibrio* or *Aeromonas* species, with a higher prevalence in warm coastal regions [1]. *Escherichia coli* (*E. coli*) is rarely identified in cases of polymicrobial NF, particularly in Fournier’s gangrene, and its occurrence in monomicrobial NF is infrequent [4]. Of note, there are few reports of monomicrobial NF caused by *E. coli* or *Klebsiella pneumoniae*, which are not currently included in the existing NF classification [1].Monomicrobial gram-negative NF, specifically involving *E. coli* or *Klebsiella pneumoniae*, is more commonly seen in elderly patients with concurrent medical conditions such as liver cirrhosis, chronic kidney disease, or other comorbidities [1]. This subset of NF is associated with poorer clinical outcomes.

Additionally, gram-negative infections typically elicit a more robust inflammatory response, characterized by elevated concentrations of inflammatory markers compared to gram-positive infections [5]. Among the monomicrobial gram-negative NF cases, *E. coli* is notable for its significantly increased mortality risk, even when adjusting for patient age and comorbidities [1].

Herein, we present a novel case of monomicrobial NF caused by *E. coli* in an otherwise healthy man.

## Case presentation

A man in his 50s with no significant past medical history presented with two days of worsening swelling, pain, and erythema in his left lower extremity. He reported no inciting event, injury, or recent trauma to the area and denied any other symptoms. On presentation, he was tachycardic, but other vital signs were stable. Physical examination revealed significant edema, erythema, warmth, and tenderness to palpation on the distal calf and anterior shin of the left lower extremity; however, there was no evidence of crepitus, fluctuance, or purulence. Skin discoloration and thickening were also noted along both lower extremities. The patient’s BMI was 53.62 kg/m^2^. Notable lab findings include a white blood cell count of 15,400 cells/μL, serum creatinine of 1.41 mg/dL, potassium of 3.1 mEq/L, erythrocyte sedimentation rate of 88 mm/hour, and C-reactive protein of 32.8 mg/L. Serum lactate was within the normal limits. A left lower extremity venous duplex ultrasound was negative for deep vein thrombosis.

**Figure 1:**
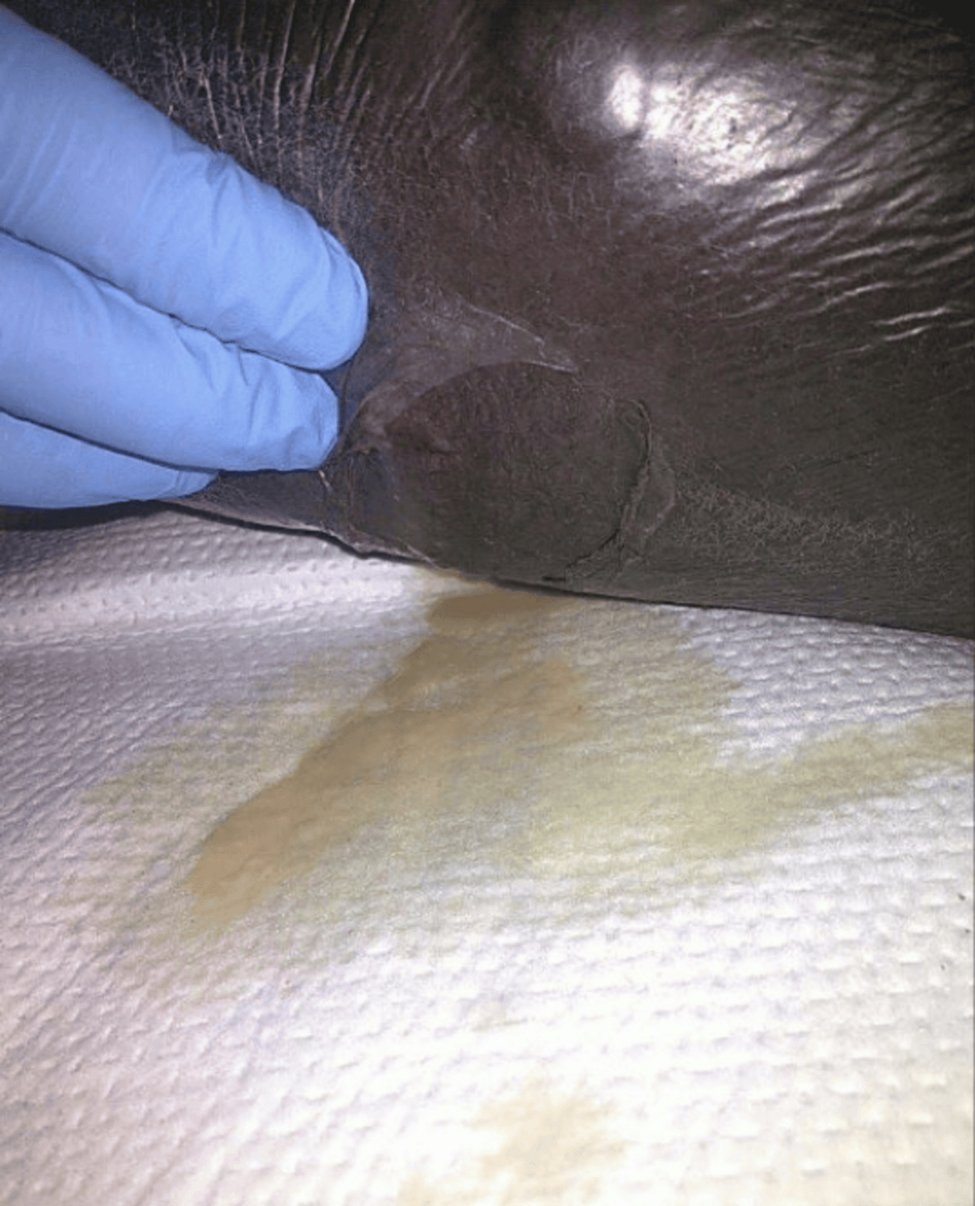
Weeping blister 3.5 x 3.5 cm on the distal calf of the left lower extremity

The patient was diagnosed with sepsis secondary to left lower extremity cellulitis. He was started on intravenous (IV) ceftriaxone and vancomycin. Blood cultures showed growth of *E. coli* in one out of two specimens; hence, antibiotic therapy was de-escalated to ceftriaxone monotherapy. CT of the left lower extremity showed diffuse soft tissue edema along the left lower extremity without fluid collection, soft tissue gas, or acute osseous abnormality. The patient subsequently developed fevers and continued leukocytosis, for which he received one dose of ertapenem while awaiting sensitivity data and 24 hours of clindamycin for toxin inhibition due to significant inflammation. As *E. coli* is not a typical pathogen for lower extremity cellulitis/abscess, an abdominal and pelvic CT was performed, which ruled out acute intra-abdominal processes. The patient’s hospital course was complicated by acute kidney injury, rhabdomyolysis, and worsening leukocytosis. The patient reported improvement in leg pain; however, his wound formed new areas of weeping and blistering (Figure 1). Antibiotics were switched to IV meropenem, and IV linezolid was added, as it was believed that the patient required reliable methicillin-resistant *Staphylococcus aureus* (MRSA) coverage for now purulent cellulitis. 

**Figure 2:**
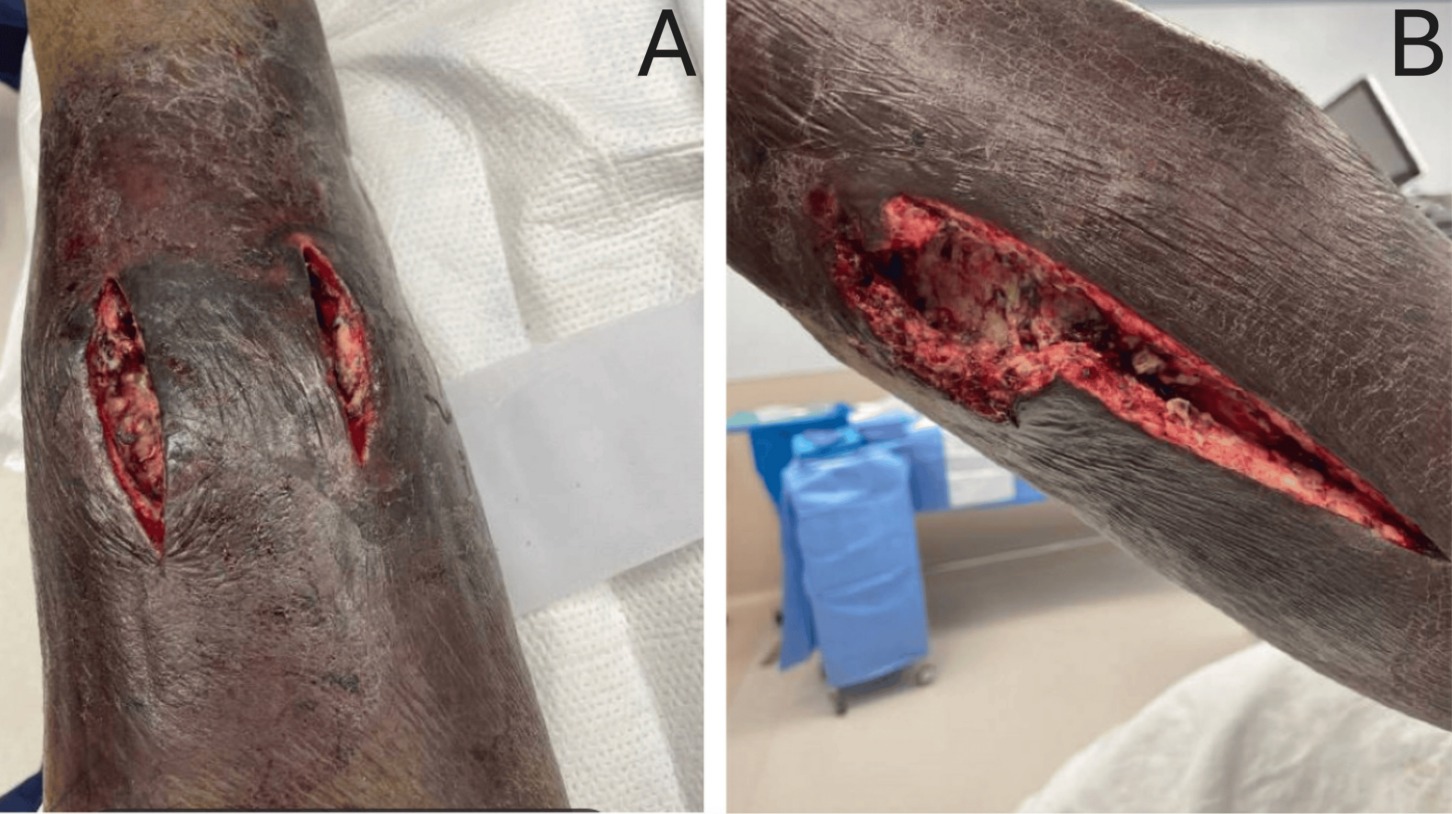
(A) Wound status post (s/p) second debridement on the anterior shin of the left lower extremity. (B) Wound s/p second debridement on the posterolateral calf of the left lower extremity

A repeat CT of the left lower extremity three days later was notable for air-fluid collection along the distal calf. The patient underwent incision and drainage of the abscess and debridement of the necrotic tissue, with four additional debridements and wound vac placement during the hospital course (Figure 2). Operative tissue cultures persistently grew *E. coli*. Antibiotics were switched to IV piperacillin-tazobactam, and the patient completed a three-week course of antibiotic therapy.

Treatment

Effective antimicrobial therapy, alongside prompt surgical debridement, is critical in reducing the mortality risks associated with NF [1]. Empirical antibiotic recommendations for NF primarily target gram-positive bacteria, typically involving the use of penicillin or ampicillin in combination with clindamycin [3,5]. However, due to the increasing incidence of gram-negative bacteria as etiological agents, as seen in this case, empirical antibiotic therapy with activity against gram-negative pathogens may need to be considered [3]. In this case, cultures grew *E. coli* resistant to ampicillin, gentamicin, and tetracycline but sensitive to ceftriaxone, doxycycline, amoxicillin-clavulanate, ertapenem, and piperacillin-tazobactam. The patient clinically improved on IV piperacillin-tazobactam.

Outcome and follow-up

Optimal survival outcomes are achieved in patients who undergo prompt radical debridement, receive adequate hydration, and are administered broad-spectrum antibiotics. Our patient did not experience additional adverse prognostic factors, such as loss of consciousness, respiratory distress, renal failure, or acute respiratory distress syndrome.

The patient reported feeling well with progressive wound closure. Given the nature of NF, most patients have extensive open wounds that necessitate frequent dressing changes over weeks or months, requiring regular wound care nurse visits.

Initially, the wound care nurse visited the patient's home three times a week for dressing changes. However, the patient resumed work five months post-discharge, prompting a reduction in nurse visits to once every Saturday.

## Discussion

To the best of our knowledge, after reviewing the PubMed literature, this is the only reported case of monobacterial *E. coli* NF in an immunocompetent individual without any skin lesions, open wounds, or trauma precipitating the disease. Li et al. presented a case of *E. coli-*associated NF in a patient with nephritic syndrome [6]. Grimaldi et al. reported a monomicrobial *E. coli* NF case in an aplastic anemia patient [7], while Shaked et al. documented a series of seven cases involving flesh-eating *E. coli* strains carrying the CNF1 toxin gene, all resulting in fatalities, with three deaths occurring within 48 hours of admission [4]. All the patients had chronic conditions, including malignancy, chronic liver disease, and renal failure [3]. However, our patient was immunocompetent. We could not identify any predisposing factors leading to *E. coli* infection, including prior administration of antibiotics covering gram-positive organisms before the clinical presentation. 

The occurrence of *E. coli* as a monomicrobial pathogen leading to NF is exceedingly rare in the literature, with reported incidences ranging from 0% to 10% [4]. Our case presentation emphasizes that the elevated mortality risk associated with type 3 NF cannot be solely attributed to other comorbidities [1]. It has been hypothesized that the presence of extraintestinal virulence factors facilitates the hematogenous spread of extraintestinal *E. coli* strains [1], as evidenced by cases like the patient reported here, who presented with bacteremia. Gram-negative infections are associated with a higher incidence of organ dysfunction related to sepsis or septic shock. The presence of sepsis at admission signals an unfavorable outcome in patients with lower extremity NF [5].

## Conclusions

Physicians need to be aware that NF can occur solely from *E. coli* infection. In addition to targeting gram-positive organisms, empirical antibiotic therapy with coverage against gram-negative organisms should be considered. Further research and investigation are necessary to develop strategies to mitigate and prevent the spread of this severe infection.
